# The Effect of Stress among Medical Representatives Working in Coimbatore City, India

**Published:** 2019-08

**Authors:** Priya KALYANASUNDARAM

**Affiliations:** Sankara Institute of Management Science, Saravanampatty, Coimbatore, India

## Dear Editor-in-Chief

The purpose of this study was to understand the impact of stress among medical representatives in Coimbatore City, India. Pharmaceutical sales representatives play a large role in helping an industry in a rapidly changing environment to achieve a new corporate vision and normally these medical representatives are in a highest stress position. Occupational stress index was given to assess the stress levels. The study found that organisation can reduce role conflict & role ambiguity by adopting a specific role strategy and the expectations of the medical representatives to be compared with their actual roles job profile and working hours need to be considered in the context of the well- being of the medical representatives.

Pharmaceutical Companies are a typical marketing industry dealing with mainly medical professionals, for marketing different pharmaceutical products companies require more and more skilled competent representatives to develop good rapport with their direct customer. The workplace for medical representatives has become a high stress environment in many organizations cutting across industries. Hence it was felt pertinent to study the effect of stress among medical representatives in Coimbatore city, Tamilnadu, India.

Stress as “it is the consequence that an individual’s ability or skill fails to coordinate with the job or the job environment can not satisfy the individual demand” (1). Defined stress as the general term that refers to any demand psychological or physical that is outside the norm (2). Defines stress as the reactions of body to negative influences (3) “Stress is the sum of all non-specific changes caused by function or damage” (4).

Our aims of the study were as follows:

1. To study the effect of stress on medical representatives.
2. To analyse the stress in medical representatives and its impact on them.
3. To identify the level of occupational stress among the employees.
4. To suggest suitable measures to overcome the drawbacks.

We chose Coimbatore as an area for study as Coimbatore is the second biggest city of the southern state of Tamil Nadu. The population comprised of medical representatives working in Coimbatore City.

Occupational Stress Index scale (5) was administered used to measure the Occupational Stress of the respondents. The scale consists of 12 factors having 46 items each rated on the five –point scale. This scale aims at measuring the extent of stress, which the employees perceive arising from various constituents and conditions of their job. The response options ranged from strongly disagree to Strongly Agree (on a rating scale of 1 to 5).

Similarly in case of medical representatives, details and particulars regarding the number of medical representatives were obtained from the area managers. There were 1380 medical representatives then. We adopted systematic simple random sampling method for selecting the sample, the names of medical representatives were arranged in alphabetical order and from that list every 2^nd^ respondent of the population starting from random number two was chosen. At the end of the data collection there were 552 qualified filled in respondent.

We personally administered the questionnaires for managers and medical representatives. We relied on electronic data EBSCO, doctoral thesis, academic journals, internet and books.

The results are presented in Table 1.

**Table 1:** The correlation between stress factors

***Variable***	***RO***	***RA***	***RC***	***UGP***	***ROP***	***UP***	***PLN***	***PPR***	***IM***	***LS***	***SWC***	***UNP***	***TS***
RO	1												
RA	.506(**)	1											
RC	.468(**)	.089(*)	1										
UGP	.420(**)	.150(**)	.017	1									
ROP	.467(**)	.121(**)	.136(**)	.087(*)	1								
UP	.280(**)	.124(**)	.075	.039	.107(*)	1							
PLN	.433(**)	.661(**)	.036	.062	.111(**)	.114(**)	1						
PPR	.368(**)	.016	.603(**)	.001	.074	.100(*)	.075	1					
IM	.370(**)	.031	−.007	.690(**)	.070	.018	.099(*)	.111(**)	1				
LS	.256(**)	−.035	−.066	−.018	.502(**)	−.021	.036	.063	.113(**)	1			
SWC	.660(**)	.303(**)	.284(**)	.299(**)	.323(**)	.541(**)	.478(**)	.554(**)	.472(**)	.389(**)	1		
UNP	.658(**)	.292(**)	.295(**)	.318(**)	.338(**)	.185(**)	.516(**)	.619(**)	.559(**)	.493(**)	.898(**)	1	
TS	.864(**)	.474(**)	.438(**)	.429(**)	.460(**)	.469(**)	.504(**)	.527(**)	.470 **)	.337(**)	.899(**)	.854(**)	1

**RO -** *Role Overload,* **RA -** *Role Ambiguity,* **RC -** *Role Conflict,* **UGP -** *Unreasonable Group Pressure,* **ROP –** *Responsibility Of Persons,* **UP -** *Under-participation,* **PLN –** *Powerlessness,* **PPR -** *Poor Peer Relations,* **IM -** *Intrinsic Improvisation,* **LS -** *Low Status,* **SWC -** *Strenuous Work Conditions,* **UNP –** *Unprofitability***, TS –** *Total Stress*

Policies regarding their travelling can be framed, as they have to travel a lot, which increase the accidental risk while travelling. Meditation, yoga therapy and counselling can be incorporated to reduce work related stress. Management can reduce role conflict & role ambiguity by adopting a specific role strategy and the expectations of the medical representative to be compared with their actual roles. Development in the nature of job profile and working hours need to be considered in the context of the well- being of the medical representatives. Stress audit to be undertaken to identify ‘stress areas’ in order to improve condition of job and alleviate job stress.

Today, Pharmaceutical companies are going global through exports, joint ventures, mergers and acquisitions, and out-licensing, tasks cannot simply be accomplished individually or by working with others in fixed mundane or routine ways. Hence the organisations has to focus towards developing the behaviour of the employees, so that they can be competitive as it is very essential that medical representatives need diverse set of skills which can make business profitable. As stress affects the employees’ performance that indirectly affects the organization survival. The organization should develop suitable strategies to reduce among medical representatives so that in the long run it will contribute towards organization’s success.
